# A Typical Aortic Intramural Hematoma With an Atypical Neurological Presentation

**DOI:** 10.7759/cureus.48696

**Published:** 2023-11-12

**Authors:** Shazaf M Sidhu, Muhammad Daniyal Waheed, Sadaf Sheikh, Tabseer Ahmed, Mariam Nasir

**Affiliations:** 1 Internal Medicine, Jinnah Medical and Dental College, Karachi, PAK; 2 Internal Medicine, Foundation University Medical College, Islamabad, PAK; 3 Emergency Medicine, Sultan Qaboos University Hospital, Muscat, OMN

**Keywords:** tevar, acute aortic syndromes, atypical neurological presentation, spinal cord infarction, aortic intramural hematoma

## Abstract

Aortic intramural hematoma (IMH) is characterized by blood spilling through the intimal layer of the aortic wall without any tear within the aortic wall. The condition has been troublesome to analyze until of late. A 55-year-old gentleman with hypertension presented with epigastric pain radiating to the back, he later developed back pain as well as bilateral lower limb numbness and was found to have IMH when a CT angiogram was conducted. Due to the severity of the illness, the patient expired on the 10th day of the admission. It is important for physicians to be aware of atypical presentations of this life-threatening aortic disease.

## Introduction

Acute aortic syndrome (AAS) is a group of life-threatening aortic diseases that encompasses aortic dissection (AD), intramural hematoma (IMH) and penetrating aortic ulcer. In spite of the fact that this ailment is comparative to, and was considered to be a subset of AD, the restorative circle presently considers aortic IMH to be different as there is no tear within the wall of the aorta [1-3]. Its natural history is inconsistent; it can either resolve without any intervention, or it may progress to typical AD with 15-20% of patients having an outward aortic rupture [2]. Approximately 10-30% of patients with AAS have IMH. Multidetector computed tomography (CT) is the foremost technique for diagnosing and classifying IMH; however, MRI is brought into play for serial follow-up studies [4]. IMH on radiological imaging is defined as the presence of aortic wall thickness of greater than 7 mm without intimal disruptions, a dissecting membrane and false lumen flow [4]. 

Acute thoracic back pain or chest pain is the classical presentation of AAS but can be associated with many neurological symptoms which include seizures, stroke, Horner’s syndrome and coma [5,6]. Spinal cord infarction (SCI) is a very rare neurological presentation of IMH that has been published in a handful of case reports [3,5,7]. We report a case of an extremely rare presentation of SCI caused by IMH in a tertiary care hospital.

## Case presentation

**Figure 1:**
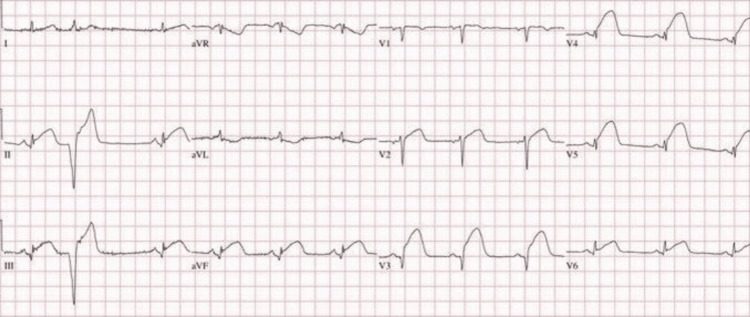
ECG of the patient showing ST elevation in precordial leads

A 55-year-old gentleman, with hypertension for 15 years and well compliant on medications, presented in the emergency department with epigastric pain and burning. The pain was radiating to his back. On arrival, his blood pressure was recorded as 130/90 mm Hg with no change in blood pressure from both arms. His heart rate was 80 beats per minute and oxygen saturation was 99% on room air. The electrocardiogram showed normal sinus rhythm. The blood investigations of the patient including troponin and amylase were unremarkable. Within the next 30 minutes, he mentioned lower back pain with bilateral numbness of his lower limbs. His repeat electrocardiogram showed ST elevation in precordial leads (Figure 1).

**Figure 2:**
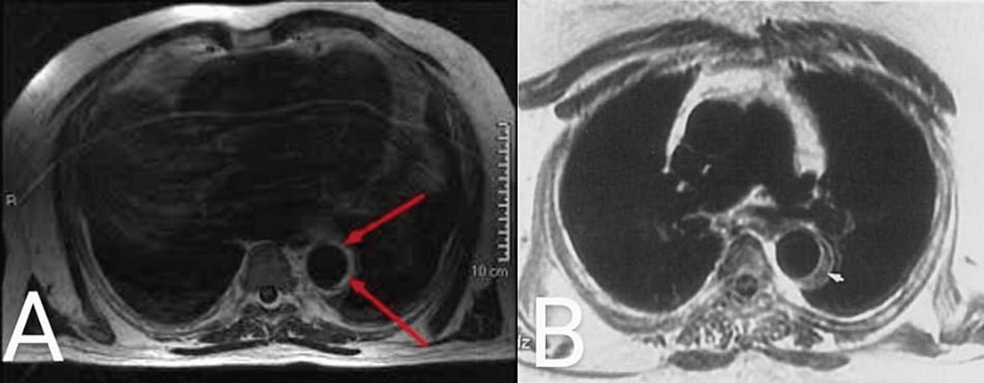
MRI showing increased signal intensity with diffusion restriction (A) T1-weighted image of cardiac MRI depicting blood flow in the descending aorta which is surrounded by the rim of bright material with increased echogenicity (red arrows), representing a subacute intramural hematoma. (B) T1-weighted cardiac MRI representing an intramural hematoma, surrounded with mixed hypo- and isodense rim (white cursor) suggesting a chronic duration.

Cardiology was taken on board and an urgent diagnostic coronary angiogram was performed. The angiography via the right radial artery showed no findings of any obstructive coronary artery disease and the invasive aortogram with runoff revealed no narrowing typical for aortic aneurysm. MRI of the spine was advised to rule out cord compression, which showed increased T2 signal intensity with diffusion restriction that mainly involved the central grey matter of the spinal cord extending from the T4 to T11 level (Figure 2A, 2B).

**Figure 3:**
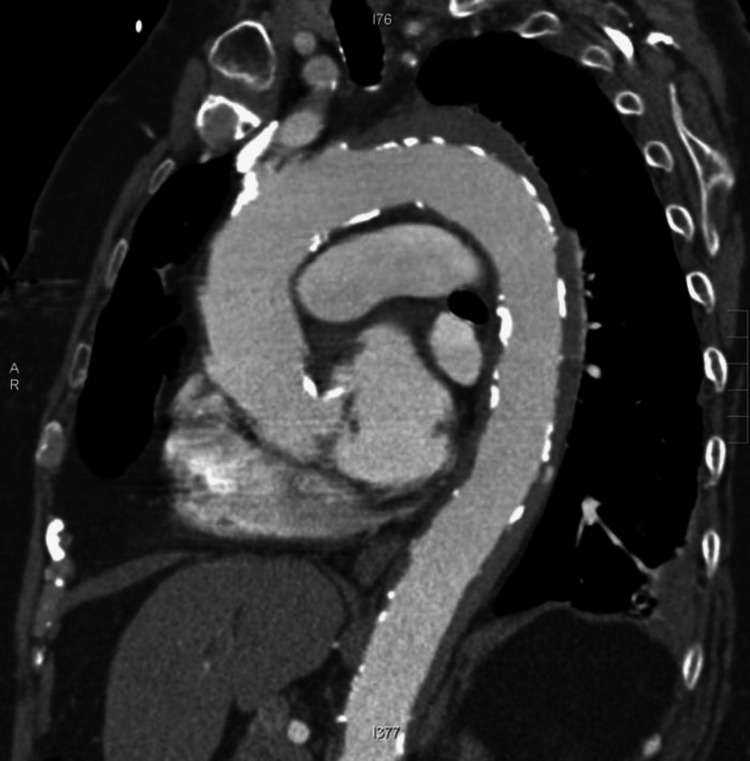
CT axial showing aortic intramural hematoma

The computed tomography angiogram (CTA) indicated a small volume of pericardial effusion along with acute IMH extending from the ascending aorta to the suprarenal aorta without any extravasation of contrast, great vessel occlusion or hemothorax. His mental status deteriorated progressively without developing manifestations in other organs (Figure 3). He was managed in an intensive care unit where he expired on the 10th day of admission. His leading cause of death was cardiac tamponade secondary to IMH with spinal cord infarct.

## Discussion

Although IMH is rare, it is a life-threatening disease of the aorta. It is most commonly diagnosed in elder men between the ages of 60 and 80 years [8-10]. It occurs in an area where the aorta is weakened; this can occur as a consequence of hypertension, atherosclerosis, Marfan syndrome, Takayasu's arteritis, prior aortic surgery, aortic aneurysm, coarctation of the aorta, bicuspid aortic valve, pregnancy and prior cardiac surgery [2].

The classical presentations of AAS/IMH are acute chest pain and thoracic back pain [5]. Neurological presentations at the onset of AAS are few and far in between but are generally dramatic, obscuring the primary vascular problems [7]. The most common neurological symptoms include seizures, stroke, Horner’s syndrome and coma [5,6].

SCI is an extremely rare neurological presentation of IMH [5]. The symptoms vary from mild weakness to paraplegia or paraparesis on the first encounter [5,7]. SCI occurs in only 2% of all neurological vascular diseases [11]. While in cases of AAS, SCI has an occurrence of only 2-9% [5]. In fact, the prevalence of SCI due to IMH was so rare that Tsushima T et al. were able to find only seven case reports in the last decade during their study on atypical complications of aortic IMH [7].

IMH can be classified as Stanford type A involving the ascending aorta and Stanford type B involving the aortic arch or descending aorta [6]. Type A IMH is complicated by hemopericardium and acute aortic regurgitation. 3.3% of IMH involve coronary arteries presenting as ST-elevation myocardial infarction leading to unfruitful cardiac catheterization and heparinization [12,13]. Involvement of the ascending aorta along with an aortic diameter of more than 50 mm is a critical predictor of increased mortality in IMH [3,14]. IMH may progress into AD over time in 47% of cases but they may also regress spontaneously or remain static [7,14]. Acute aortic IMH masked by neurological features such as SCI, which is extremely rare, worsens the prognosis due to the delay in diagnosis, lack of management strategies and a short therapeutic window [7]. In well-established tertiary care setups, emergency surgical measures may provide a better outcome than medical treatment for patients with acute type A aortic IMH [4,12,13] while aggressive management of hypertension showed good short-term outcomes in patients with Stanford type B IMH [4]. Thoracic endovascular aortic repair (TEVAR), a less invasive procedure, has also come into play as an alternative to open surgery for the treatment of type B AAD and ascending AAS [6,7]. A fruitful outcome of all these treatment approaches is only possible if the physician can correlate the symptoms of SCI, lab and imaging findings and diagnose IMH timely in the specific therapeutic window.

## Conclusions

Neurological complications with AASs are frequent and occur in 17-40% of patients, but SCI is extremely rare and very rarely lands in any tertiary care setup. It is imperative to keep IMH in mind with any patient presenting with typical chest or epigastric pain along with neurological features - an IMH can surprise with atypical neurological features.
